# Supplementary material for: Role and mechanism of NCAPD3 in promoting malignant behaviors in gastric cancer
Source: Front Pharmacol. 2024 Apr 22;15:1341039. doi: 10.3389/fphar.2024.1341039 (PMC11070777; doi:10.3389/fphar.2024.1341039)
Supplement: Supplementary file 11 [file DataSheet2.ZIP › GSEA/immunologic/my_analysis.Gsea.1599462003817/GSE411_UNSTIM_VS_100MIN_IL6_STIM_SOCS3_KO_MACROPHAGE_DN.html]

Details for gene set GSE411\_UNSTIM\_VS\_100MIN\_IL6\_STIM\_SOCS3\_KO\_MACROPHAGE\_DN[GSEA]

|  || Dataset | filtered\_dataset.sample\_info.cls#WT\_versus\_NCAPD3\_MUT |
| Phenotype | sample\_info.cls#WT\_versus\_NCAPD3\_MUT |
| Upregulated in class | NCAPD3\_MUT |
| GeneSet | GSE411\_UNSTIM\_VS\_100MIN\_IL6\_STIM\_SOCS3\_KO\_MACROPHAGE\_DN |
| Enrichment Score (ES) | -0.55244255 |
| Normalized Enrichment Score (NES) | -2.496145 |
| Nominal p-value | 0.0 |
| FDR q-value | 0.015132694 |
| FWER p-Value | 0.116 |
Table: GSEA Results Summary

  

Fig 1: Enrichment plot: GSE411\_UNSTIM\_VS\_100MIN\_IL6\_STIM\_SOCS3\_KO\_MACROPHAGE\_DN      
 Profile of the Running ES Score & Positions of GeneSet Members on the Rank Ordered List

  

| SYMBOL | TITLE | RANK IN GENE LIST | RANK METRIC SCORE | RUNNING ES | CORE ENRICHMENT || 1 | 3188 | HNRNPH2 | 769 | 0.338 | -0.5102 | No |
| 2 | 5898 | RALA | 813 | 0.309 | -0.5025 | No |
| 3 | 50640 | PNPLA8 | 881 | -0.296 | -0.5136 | Yes |
| 4 | 2150 | F2RL1 | 887 | -0.304 | -0.4792 | Yes |
| 5 | 55971 | BAIAP2L1 | 906 | -0.333 | -0.4505 | Yes |
| 6 | 27131 | SNX5 | 907 | -0.333 | -0.4088 | Yes |
| 7 | 3949 | LDLR | 929 | -0.353 | -0.3798 | Yes |
| 8 | 9388 | LIPG | 937 | -0.361 | -0.3397 | Yes |
| 9 | 1978 | EIF4EBP1 | 938 | -0.362 | -0.2944 | Yes |
| 10 | 10123 | ARL4C | 957 | -0.377 | -0.2603 | Yes |
| 11 | 83641 | FAM107B | 960 | -0.381 | -0.2141 | Yes |
| 12 | 56261 | GPCPD1 | 993 | -0.400 | -0.1871 | Yes |
| 13 | 91663 | MYADM | 1004 | -0.407 | -0.1433 | Yes |
| 14 | 10018 | BCL2L11 | 1027 | -0.425 | -0.1060 | Yes |
| 15 | 6590 | SLPI | 1062 | -0.453 | -0.0738 | Yes |
| 16 | 8444 | DYRK3 | 1131 | -0.502 | -0.0599 | Yes |
| 17 | 9709 | HERPUD1 | 1202 | -0.578 | -0.0380 | Yes |
| 18 | 57533 | TBC1D14 | 1304 | -0.708 | -0.0220 | Yes |
| 19 | 1649 | DDIT3 | 1341 | -0.779 | 0.0496 | Yes |
Table: GSEA details [plain text format]

  

Fig 2: GSE411\_UNSTIM\_VS\_100MIN\_IL6\_STIM\_SOCS3\_KO\_MACROPHAGE\_DN      
 Blue-Pink O' Gram in the Space of the Analyzed GeneSet

  

Fig 3: GSE411\_UNSTIM\_VS\_100MIN\_IL6\_STIM\_SOCS3\_KO\_MACROPHAGE\_DN: Random ES distribution      
 Gene set null distribution of ES for **GSE411\_UNSTIM\_VS\_100MIN\_IL6\_STIM\_SOCS3\_KO\_MACROPHAGE\_DN**
